# Neutrophil-to-Lymphocyte Ratio as a Prognostic Marker of Disease Severity in Community-Acquired Pneumonia Among Hospitalized Patients From Northeast India

**DOI:** 10.7759/cureus.98524

**Published:** 2025-12-05

**Authors:** Abhisek Ghosh, Aritra Banerjee, Pranjeeta Deka, Deepak K Pandey, Dibya J Sharma, Akash Batta

**Affiliations:** 1 General Medicine, Silchar Medical College and Hospital, Silchar, IND; 2 Medicine, Silchar Medical College and Hospital, Silchar, IND; 3 Internal Medicine/Gastroenterology, Silchar Medical College and Hospital, Silchar, IND; 4 Cardiology, Dayanand Medical College and Hospital, Ludhiana , IND

**Keywords:** biomarker, community-acquired pneumonia (cap), curb-65 score, medical icu, mortality, neutrophil-to-lymphocyte ratio (nlr), prognosis, severity

## Abstract

Background: The role of the neutrophil-to-lymphocyte ratio (NLR) as a prognostic marker for community-acquired pneumonia (CAP) has not been extensively studied in India. Therefore, we conducted this study to assess the role of the NLR in determining prognosis and severity among hospitalized CAP patients from Northeast India.

Materials and methods: This prospective observational study was conducted from October 2023 to November 2024 in a tertiary care hospital in southern Assam, Northeast India. A total of 162 adult patients with CAP were recruited for the study via consecutive sampling. Statistical analysis included descriptive statistics, chi-square tests, correlation analysis, and logistic regression to evaluate associations between NLR, severity scores (CURB-65), and patient outcomes.

Results: A strong positive correlation (r = 0.72) was noted between the NLR and CURB-65 score. A level of NLR ≥ 9.5 anticipated the need for intensive care unit (ICU) admission (40.74%, n=66 out of 162), while NLR ≥ 16 was associated with a higher death rate (80%, n=16; out of 20 deaths) compared to NLR <16 (20%, n=4). Most patients (55%) were middle-aged men. Right middle zone pneumonia (22.8%) was the most frequent radiological finding, while 85.18% patients had sterile sputum culture. Both Klebsiella pneumoniae (6.2%) and Streptococcus pneumoniae (6.2%) were the most typical organisms noted in the culture specimen.

Conclusion: An elevated NLR is a strong predictor of disease severity, ICU requirement and mortality amongst Indian CAP patients.

## Introduction

Reliable prognostic assessment of community-acquired pneumonia (CAP) is decisive for ensuring patient safety and guiding optimal management [1]. Various mechanisms, including clinical, imaging, and biomarker-based mechanisms, exist to aid in this prediction. Most current clinical severity systems, like CURB-65 (consisting of confusion, blood urea nitrogen (BUN) >7 mmol/L, respiratory rate (RR) >30/min, systolic blood pressure (SBP) < 90 mmHg and/or diastolic ≤ 60 mmHg and age ≥65 years) [1,2], Pneumonia Severity Index (PSI score comprising 20 different demographic as well as clinical factors, categorizing patients into five risk classes, from Class I (lesser risk) to Class V ( most hazardous)), [3] and SMART-COP (includes SBP, multilobar infiltrates, serum albumin levels, respiratory rate, tachycardia, confusion, oxygen saturation and pH) which include a mix of clinical, laboratory and radiographic findings [4,5]. Simultaneously, new prognostic tools like the Severe Community-Acquired Pneumonia (SCAP) score [6], expanded CURB (eCURB) system [7], and AI/ML (artificial intelligence/machine learning) tools are being developed for a higher predictive accuracy and reliability [8]. However, gaps in predictive accuracy still exist, especially in the applicability of the present systems amongst a diverse spectrum of patients. Other challenges of the present systems include variability, lack of specificity, validity, and cost-effectiveness [5].

Biomarkers are increasingly considered important adjuncts in improving the diagnosis of disease [7,8]. These include the markers of inflammation and infection leading to immune response, tissue injury, and malfunction [9]. They may help in risk stratification of CAP more accurately, enhancing early prognosis and facilitation of treatment decisions. The most common and proposed markers for CAP are C-reactive protein (CRP) [9], procalcitonin (PCT) [10], and neutrophil-to-lymphocyte ratio (NLR) [11]. Other biomarkers, including serum lactate [12], interleukin-6 (IL-6) [13], pro-adrenomedullin (pro-ADM), B-type natriuretic peptide (BNP)/pro-BNP [14], D-dimer [15], and troponin, are also being increasingly explored in CAP [16].

Among these, the NLR has garnered a decent amount of research interest of late [17]. The reason is primarily due to good predictive CAP severity outcomes, as well as accessibility and cost-effectiveness [11,17]. Since its use as a diagnostic adjunct and a biomarker for risk stratification in early waves of COVID-19, use of the NLR as a prognostic biomarker is increasingly considered for other pulmonary diseases, especially for CAP [18].

The NLR quantitatively reflects the balance between innate and adaptive immune responses, the two important components of the host defence system during CAP [11]. Development of CAP leads to a systemic inflammatory response wherein neutrophils act as first responders. Therefore, neutrophilia suggests a dysregulated inflammatory response associated with severe CAP or bacterial superinfection [11]. Additionally, lymphopenia associated with CAP may indicate immunosuppression and a pointer towards unfavourable consequences, especially in vulnerable population groups such as the elderly [11]. Therefore, a high neutrophil lymphocyte ratio indicates a strong inflammatory response, poor immune regulation, greater disease severity, and poorer prognosis for CAP [11].

The existing literature reinforces the potency of the NLR as a prognostic adjunct. However, there seems to be an ambiguity in the prognostic cut-offs to guide effective clinical decisions. Furthermore, there is a dearth of literature about the prognostic capability of NLR in the Indian context, more so from the North-eastern region. Therefore, this study was conducted to appraise the role of the NLR in predicting the severity and outcome of CAP in the Indian scenario.

## Materials and methods

The index study has been accomplished in accordance with the STROBE (Strengthening the Reporting of OBservational studies in Epidemiology) statement.

Study design and setting

This prospective hospital-based observational study was undertaken over one year in the Department of General Medicine, Silchar Medical College and Hospital, Silchar, Assam, a tertiary care medical institution in the north north-eastern part of India. Patients were enrolled using a consecutive sampling method. All eligible patients presenting during the study period who met the inclusion criteria were recruited consecutively. 

Patient recruitment (eligibility, sampling, consent)

Adults (≥18 years) who were hospitalized with a clinical diagnosis of CAP were included. Written informed consent was taken in the format approved by the institutional ethics committee from all the participants. Approval for the study was obtained by the institutional ethics committee vide letter no SMC/ETHICS/M2/2025/25. A standard institutional protocol was followed for the treatment of CAP patients in our study. 

Inclusion criteria

Patients with radiographic findings on chest X-ray (CXR) and/or CT (computed tomography) Chest with contrast of the thoracic region in addition to the clinical presentation that is typical of CAP were included. CAP was diagnosed as "acute infection involving lung parenchyma, presenting with fever, cough with or without expectoration, breathlessness, pleuritic chest pain, along with radiological imaging depicting new infiltrate or consolidation which is not caused by other possible pulmonary causes. The infection needs to be acquired outside a hospital in a patient who is neither hospitalized nor received health care facility treatment in the preceding 14 days" [19].

Exclusion criteria

In this prospective study, patients who denied informed consent, immunocompromised individuals, pregnant women, patients with aspiration pneumonia, chemical pneumonitis, hospital-acquired pneumonia, or ventilator-associated pneumonia were excluded from the study. We ruled out aspiration pneumonia using tools like Mini-Mental State Examination (MMSE), by which alertness among the patients was identified. Also, patients with an altered level of consciousness with Glasgow Coma Scale (GCS) < 15 and with known neurological conditions predisposing to aspiration (e.g. stroke, parkinsonism and dementia), documented dysphagia and choking episodes, history of recent vomiting with seen aspiration, malignancies and structural defects involving head and neck and patients who underwent procedures like tube feeding and tracheostomy were excluded. Patients with a history of dysphagia (assessed by water swallowing test) were excluded from the study as it predisposes to aspiration pneumonia. Sampling was done by a random method. Informed consent in written format was taken from all participants (or a legally authorized representative when required).

Data collection

The demographics, in-depth history, and examination were documented in a standardized proforma along with the laboratory and radiology results. Blood was analyzed for the hemoglobin level, total leukocyte count (TLC), absolute neutrophil and lymphocyte count, NLR, random blood sugar (RBS), serum creatinine, BUN, erythrocyte sedimentation rate (ESR), and CRP. Sputum examination was carried out in all patients with CAP. Localization of infiltrates was done based on imaging like chest X-ray (CXR). As per institutional protocol, all patients underwent a standard posteroanterior chest X-ray (1 view) as the first-line radiological investigation. Computed tomography (CT) of the thorax with contrast was performed based on clinical discretion and in situations such as diagnostic uncertainty, suspicion of complications like lung abscess, necrotizing pneumonia, or empyema as well as poor correlation between clinical and radiologic findings. Follow-up of the patients was conducted until the time of discharge from the hospital. The outcome (discharge or death) was observed, and the laboratory parameters were analyzed using an automated analyzer within the hospital setup.

Severity scoring tools

The PSI and CURB-65 scores were used to determine the intensity of disease among patients with CAP. The CURB-65 score was used as put forward by Lim et al. in 2003 and was subsequently adopted as a severity assessment tool for CAP in adults [2]. Similarly, the PSI/PORT (Patient Outcomes Research Team score) for CAP was applied as presented by Fine et al. [3]. Both tools are available for open access and permitted for non-commercial academic use. They are publicly accessible as already published clinical prediction regulations.

Sample size calculation

A total of 162 participants were enrolled, based on the single-proportion formula - N = Z² × p × (1 - p) / d², where p = 0.12 (estimated prevalence of CAP in hospitalized adults), d = 0.05 (precision), and Z = 1.96 (for 95% confidence level). This widely used approach, originally outlined by Charan and Biswas (2013), was adopted to ensure an adequate sample size for reliable statistical inference [20-22].

Statistical analysis

All quantitative data were analyzed using appropriate statistical methods. Analyses were performed using IBM SPSS Statistics for Windows, Version 20 (Released 2011; IBM Corp., Armonk, New York, United States). Continuous variables were summarized as mean ± standard deviation (SD) when normally distributed, and as median with interquartile range (IQR) when non-normally distributed. Categorical variables were expressed as counts and percentages.

Student’s t-test was used to compare mean values of normally distributed continuous parameters (laboratory parameters such as NLR, hemoglobin (Hb), and serum creatinine) between two outcome-based groups and ICU admissions and need outcome. A scatter plot and regression analysis were used to compare the NLR and CURB 65 score.

Ethical clearance

The written permission for the study was obtained prior to the study from the Institutional Ethics Committee of Silchar Medical College and Hospital (Approval No.: SMC/ETHICS/M2/2023/25) in October in the year 2023. The research was carried out in accordance with the institutional and national ethical policies/guidelines.

## Results

In this study, the mean age of the patients was 54.85±16.84 years. The majority of the study participants belong to the sixth decade (29.01%), followed by patients belonging to the geriatric age group (22.22%, 61-70 years). Men constituted the bulk of the patients, accounting for 55% of the total sample. The most usual length of hospital stay was five days (19.75% patients), while 11.11% of the patients had nine days of hospitalization. A significant portion of patients had mild to moderate disease severity, as reflected by the CURB-65 scores of 1 (27.78%) and 2 (25.31%), respectively. A CURB-65 score of 3 or higher, which suggests severe pneumonia and a higher risk of mortality, was observed among 20.37% (score 3), 6.17% (score 4), and 0.62% (score 5) of the patients, respectively. The relationship between CURB-65 scores and study outcomes was also analyzed. Among 20 patients who died, 40% (n=8) of the cases had a CURB-65 score of 4, while 45% (n=9) patients had a score of 3. The majority of the patients with a CURB-65 score of 0, 1, or 2 (72.8% of the cases) recovered, whereas those with scores >2 had a higher mortality risk (27.16% cases). A higher proportion of patients with severe CURB-65 scores (≥ 3) required ICU admission and ventilatory support compared with those having lower scores, and this association was statistically significant (p < 0.001, χ² test). These findings indicate that higher CURB-65 scores are linked with increased mortality, whereas lower scores are more commonly linked with favorable outcomes as shown in Table 1.

**Table 1:** Baseline demographic and clinical characteristics of patients with community-acquired pneumonia (CAP) The table summarizes age, gender, hospital stay, CURB-65 categories, ICU admission, and ventilatory support among 162 patients. Data are shown as frequency and percentage. The χ² test was used to assess associations between CURB-65 severity and clinical outcomes.

Variable	Category	Number of patients	Percentage (%)
Age (years) (Mean age approx. 54 years)	≤ 20	8	4.94
	21 – 30	5	5.56
	31 – 40	17	10.49
	41 – 50	15	9.26
	51 – 60	47	29.01
	61 – 70	36	22.22
	>70	30	18.52
Gender	Females	73	45.06
	Males	89	54.94
Hospital stay (days) (Mean hospital stay 6.66 days)	≤5 days	73	45.06
	≥5 days	89	54.94
CURB-65 score	0 – 2	118	73.0
	≥3	44	27.0
ICU admission	NO	76	46.91
	YES	86	53.04
Need for mechanical ventilation	NO	118	72.84
	YES	44	27.16

The Kruskal-Wallis test was applied for continuous variables that did not follow a normal distribution, including ESR, serum urea, and RBS levels, when compared across CURB-65 categories. Associations between categorical variables, such as CURB-65 score, ICU admission, and patient outcomes, were tested using the chi-square (χ²) test in Tables 1, 2. A strong positive correlation (r = 0.72) was noted between the NLR and CURB-65 score. An increased need for ICU admission (40.74%, n=66 out of 162) was noted among patients with an NLR level of ≥ 9.5 as the rest of the patients with a lower NLR value did not require ICU admission. Similarly, NLR value ≥ 16 among CAP patients was associated with a higher death rate (80%, n=16; out of 20 deaths) as compared to NLR <16 (20%, n=4).

**Table 2:** Radiologic and microbiologic profile by disease severity (CURB-65 <3 versus ≥3) The table compares radiologic and microbiologic variables between mild/moderate and severe CAP groups (CURB-65<3 Versus CURB-65 ≥3). Categorical differences were evaluated using the chi-square test (mentioned as χ², represented as *), highlighting significant bilateral involvement and culture positivity trends in severe cases. CAP: Community-acquired pneumonia

Variable	CURB-65 <3 (%)	CURB-65 ≥3 (%)	X2 (Chi-square value)	p-value
Radiologic zone involvement				
Unilateral (in any one zone) (*)	89 (75.4)	23 (52.3)	X^2^ = 6.38	0.012
Bilateral involvement (*)	29 (24.6)	21 (47.7)	X^2^ = 6.38	-
Sputum culture result				
Sterile (*)	105.9 (89.0)	33 (75.0)	X^2^ = 4.30	0.038
Positive for Klebsiella pneumoniae (*)	6.5 (5.1)	4 (9.1)	X^2^ = 0.75	0.39
Positive for Streptococcus pneumoniae (*)	5 (4.2)	5 (11.4)	X^2^ = 2.56	0.11

Each row represents a variable or category, showing the frequency (n) and percentage (%) of participants within each subgroup. CURB-65 scores are distributed to indicate severity stratification at admission. CURB-65 (Confusion, BUN >7 millimol/L, respiratory rate (RR) >30/min, SBP < 90 mmHg and/or diastolic BP ≤ 60 mmHg and age ≥65 years).

Laboratory parameters

Table 3 shows the laboratory profile of patients according to disease severity. Patients with severe CAP (CURB-65 ≥ 3) had a significantly higher mean TLC, absolute neutrophil counts, and NLR compared with those with mild or moderate disease (p < 0.001). The median ESR, serum urea, and RBS were also higher among the severe group (p < 0.05, Kruskal-Wallis test). Mean hemoglobin and lymphocyte counts were significantly lower in severe cases (p < 0.01, Student’s t-test). These findings highlight a clear trend of increasing systemic inflammation and metabolic stress with higher severity scores.

**Table 3:** Comparison of laboratory parameters of the study population by disease severity (CURB-65 <3 versus ≥3) The table displays mean ± SD of hematologic and biochemical parameters (Hb, TLC, neutrophil, lymphocyte, NLR, RBS, creatinine, urea, ESR). Continuous data are presented as mean ± SD. Student’s t-test (mentioned as t, represented as #), and Kruskal–Wallis tests (mentioned as H, represented as *) were used as appropriate. Evaluated differences between mild/moderate and severe pneumonia groups. p-values indicate statistical significance for each variable. NLR: neutrophil-to-lymphocyte ratio; TLC: total leukocyte count; RBS: random blood sugar; ESR: erythrocyte sedimentation rate

Parameter	CURB-65 <3 (n = 118) Mean ± SD	CURB-65 ≥3 (n = 44) Mean ± SD	Test statistic (t/ H )	p-value
Hb (g/dL) (#)	11.05 ± 1.92	9.84 ± 2.04	t =2.19	0.031
TLC (× 10^3^/µL) (*)	14.64 ± 6.18	17.92 ± 7.11	H = 3.95	0.047
Absolute neutrophil count (10^3^/µL) (#)	12.11 ± 5.93	15.28 ± 6.78	t = 2.07	0.041
Absolute lymphocyte count (10^3^/µL) (#)	1.72 ± 0.65	1.28 ± 0.58	t = 2.27	0.026
NLR (*)	8.07 ± 4.52	14.21 ± 6.73	H = 12.62	<0.001
RBS (mg/dL) (#)	115.4 ± 24.6	120.3 ± 26.7	t = 0.86	0.39
Creatinine (mg/dL) (*)	1.18 ± 0.62	1.71 ± 0.84	H = 6.77	0.009
Urea (mg/dL) (*)	49.5 ± 26.1	61.9 ± 31.2	H = 3.52	0.06
ESR (mm/h) (*)	51.2 ± 25.3	62.8 ± 27.7	H = 4.21	0.04

The microbiologic and radiologic characteristics of patients are detailed in Table 4. A standard posterior-anterior chest X-ray (1 view) was carried out as the first radiological investigation among all the patients of CAP. CT of the thorax with contrast was performed in 72 cases (44.44 %). Tight middle-zone involvement (22.8%) was the most common radiological finding followed by multilobar and bilateral patterns. Sputum cultures were positive in 15.4% of cases (n=25), with Streptococcus pneumoniae and Klebsiella pneumoniae as the predominant isolates. However, prior antibiotic use before hospitalization or early empirical use of antibiotics after hospitalization could have led to sterile sputum culture in the majority (84.5%, n=137) of the patients. 

**Table 4:** Microbiologic and radiologic profile of the study population The table summarizes chest X-ray and CT thorax findings, highlighting the most common lung zones affected. It also lists bacterial pathogens identified in sputum cultures. Frequencies and percentages indicate the relative burden of each finding. Radiologic and microbiologic assessments provide insights into disease distribution and etiology.

CXR/CT Thorax findings	Involvement	Number of patients	Percentage %
	Bilateral pneumonia	25	15.43
	Left lower zone pneumonia	21	12.96
	Left middle zone pneumonia	19	11.73
	Left upper zone pneumonia	13	8.02
	Right lower zone pneumonia	22	13.58
	Right middle zone pneumonia	37	22.84
	Right upper zone pneumonia	25	15.43
Organism detected in sputum	Acinetobacter species	2	1.23
	Hemophilus influenzae	1	0.61
	Klebsiella pneumoniae	10	6.17
	Pseudomonas aeruginosa	1	0.61
	Staphylococcus aureus	1	0.61
	Streptococcus pneumoniae	10	6.17
	None identified (Sterile)	137	84.56

A significant association was observed between positive culture results and higher CURB-65 scores (p = 0.02, χ² test) as shown in Table 2.

Table 2 compares radiologic and microbiologic findings between mild/moderate (CURB-65 < 3) and severe (CURB-65 ≥ 3) CAP groups. Bilateral lung involvement, multilobar consolidation, and positive bacterial cultures were significantly more frequent among severe cases (all p < 0.001, χ² test). Patients with bilateral or multilobar pneumonia also showed longer hospital stays and higher rates of ICU admission. Bilateral infiltrates on chest X-ray imaging were detected more predominantly among atypical pneumonia patients (73.3%, n=22 out of 30) as compared to patients with typical pneumonia (2.3%, n=3 out of 132). These chest X-ray findings can be helpful in distinguishing typical vs. atypical etiology of CAP in resource-limited settings.

Radiological findings were based on chest X-ray or computed tomography localization.

**Figure 1:**
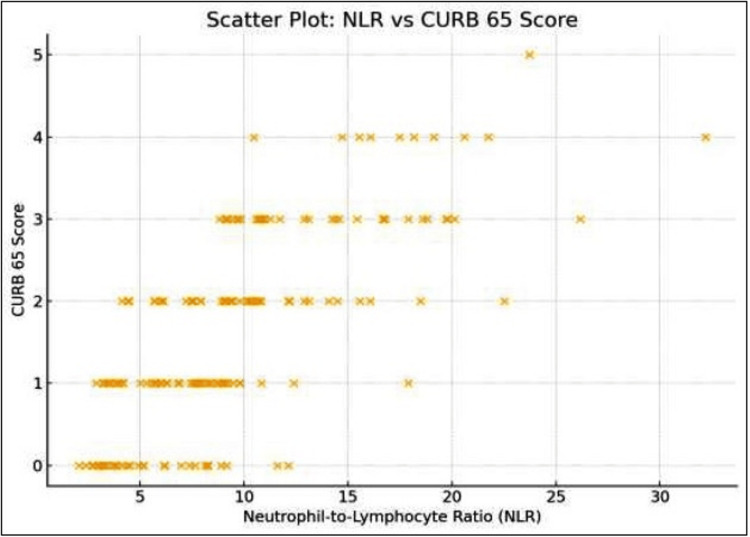
Relationship between the NLR and CURB-65 score In the figure, each data point represents one patient (n = 162). The scatter plot shows a direct association between NLR values and CURB-65 severity scores, with a positive correlation (r = 0.72, p < 0.001). The upward pattern suggests that a higher NLR corresponds to greater clinical severity of pneumonia. NLR: neutrophil-to-lymphocyte ratio

**Figure 2:**
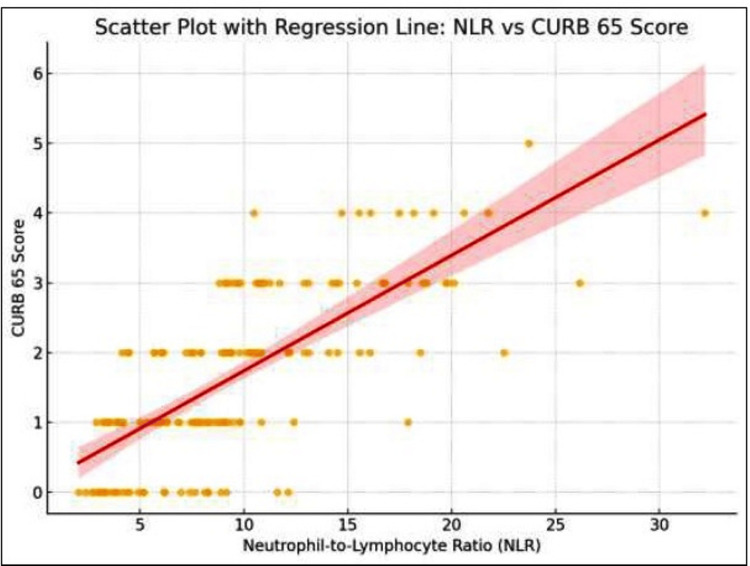
Regression analysis of the NLR and CURB-65 score The plot shown in the figure presents the same dataset as that in Figure 1 but includes a fitted regression line with 95% confidence intervals. The positive slope and narrow confidence band indicate a statistically strong association (r = 0.72, p < 0.001), emphasizing NLR’s potential as a quantitative severity marker. NLR: Neutrophil-to-lymphocyte ratio

A strong positive correlation was observed between NLR and CURB-65 score (r = 0.72, p < 0.001) and between NLR and PSI grade (r = 0.68, p < 0.001), as shown in Figure 1 and Figure 2. Correlation between NLR and CURB-65 was evaluated using Pearson’s or Spearman’s coefficients depending on data distribution, as demonstrated in Figures 1, 2. Significance was considered at a p-value of ≤ 0.05. The scatter plot (Figure 1) illustrates the upward trend in NLR values with increasing severity, while the regression plot (Figure 2) demonstrates a clear linear relationship within 95% confidence limits.

In the scatter plot, a clear upward trend was observed, reinforcing the strong positive correlation (r ≈ 0.72) and suggesting that higher NLR values are associated with greater severity as measured by the CURB-65 score as shown in Figure 2.

Figure 2 presents the same data as Figure 1 but incorporates a least-squares regression line with 95% confidence intervals. The upward slope indicates a statistically significant positive correlation between NLR and CURB-65 scores (r= 0.72, p<0.001) supporting that patients with elevated NLR values are more likely to exhibit higher disease severity based on CURB-65 criteria. This visualization highlights the usefulness of NLR as a quantitative indicator for assessing the severity of CAP. 

Outcomes and associations

The CURB-65 distribution indicated that most patients presented with mild to moderate disease (CURB-65 scores 1-2). Among the 20 patients who expired, most of them had CURB-65 scores of 3 and above. Higher CURB-65 scores demonstrated a statistically significant association with mortality (p <0.001). A positive correlation was also seen between NLR and CURB-65 (r = 0.72) indicating that increases in one variable were associated with increase in the other.

Elevated NLR values were linked to unfavorable clinical outcomes, including the need for ICU admission, ventilatory support, and increased mortality in CAP patients. These NLR cut-off values were identified based on descriptive and regression analyses and should be considered indicative rather than absolute diagnostic cut-offs. These results highlight the potential usefulness of the NLR as a simple bedside predictive marker complementing established severity indices like CURB-65 and PSI as summarized in Table 5.

**Table 5:** Indicative NLR thresholds for key clinical outcomes in CAP The table presents indicative NLR thresholds for predicting key outcomes. Patients with NLR ≥ 9.5 were significantly more likely to require ICU admission (p < 0.001), those with NLR > 10 had higher odds of needing mechanical ventilation (p = 0.002), and NLR > 16 was strongly associated with in-hospital mortality (p < 0.001). NLR: Neutrophil-to-lymphocyte ratio; CAP: community-acquired pneumonia

Outcome	NLR cut-off/indicative value
ICU admission	≥9.5
Ventilation required	>10 (indicative)
Mortality	>16

This study was able to provide certain definite cut-off ranges for key clinical outcomes from the data observed in the study participants. The cut-off or indicative NLR value of ≥ 9.5 is associated with an increased likelihood of ICU admission. All ventilated patients (27.16%) had higher NLRs compared to non-ventilated ones (72.84%). Similarly, among ICU patients, NLR > 10 appeared to be predictive of ventilation requirement. Patients with NLR score values of more than 16 had a significantly worse outcome and higher mortality as shown in Table 5.

Logistic regression (univariable and multivariable) determined independent predictors of ICU need, ventilation, and mortality, adjusting for age, gender, and CURB-65 score. Logistic regression analysis confirmed that both NLR and CURB-65 score independently predicted adverse outcomes after adjusting for age and sex. For each unit increase in NLR, the odds of ICU admission rose by 14% (OR = 1.14, 95% CI 1.07-1.22), and the odds of mortality increased by 18% (OR = 1.18, 95% CI 1.09-1.27). The CURB-65 score also showed a strong independent effect on both ICU admission (OR = 2.10, 95% CI 1.35-3.26) and mortality (OR = 2.42, 95% CI 1.52-3.86). These findings demonstrate that an elevated NLR not only correlates with established severity scores but also serves as a simple, independent prognostic marker of poor outcomes in CAP. The integration of the NLR into conventional scoring systems may therefore improve early risk stratification and clinical decision-making.

## Discussion

This is a prospective, single-center study from the Northeastern part of India, which supports the hypothesis that the NLR can be used to stratify risk in CAP easily and cheaply. The results indicate a diverse sample that included a wide range of age groups, radiographic, clinical, and laboratory findings, which ensured that we were able to study the variations in NLR and its association with CAP severity and prognosis in a diverse sample. This included varied clinical outcomes, presentation, and severity.

The study showed a strong correlation between CURB-65 scores and NLR levels in CAP. This correlation emphasizes the ability of the NLR to provide an efficient indication of disease severity and prognosis. Since the NLR is a simple and easy indicator, it could be used as an adjunct to the other prognostic tools. Feng et al. (2021) in their study observed that CURB-65, along with NLR, was an accurate in-hospital mortality predictor among elderly Chinese patients [23]. The strong correlation between CURB-65 and NLR in the present study indicates that both measures are closely related and warrant a study on their combined usage as a prognostic tool for other age groups as well. Further, our results indicate that NLR levels higher than 9.5 were associated with an increased risk of ICU admissions, while levels higher than 10 were associated with increased risk of ventilator requirement among CAP patients. Similarly, the NLR level was also found to be higher among CAP patients with poorer outcomes. This indicates that the NLR can effectively stipulate the severity of CAP among hospitalized patients and may prove to be a useful prognostic marker. The biological role of NLR in infection severity has been discussed in earlier studies, demonstrating how neutrophilia and lymphopenia reflect inflammatory activation and immune suppression, respectively [11,14]. The NLR integrates both innate and adaptive immune responses, serving as a single composite marker of immune dysregulation [11]. Similar mechanisms have been demonstrated among COVID-19-infected patients as well as other severe infections, strengthening their translational relevance [18]. 

In another study from India, Abrol et al (2024) studied the utility of NLR as a diagnostic tool for severity assessment in CAP. They have compared the NLR for differentiating between tuberculous and non-tuberculous pneumonia and have found no significant difference in the levels between both subgroups of patients. It can be said that even though NLR cannot be used as a diagnostic tool for differentiating between different types of pneumonia, it can be used as a prognostic tool. Abrol et al attributed high steroid use among the patients as their study limitation, as it was conducted during the COVID-19 pandemic [24].

Alzoubi and Khanfar (2022) conducted a meta-analysis to understand the association between NLR and mortality in CAP patients from various selected studies. The meta-analysis showed that there was a significant difference in NLR levels between survivors and non-survivors. The present study has similarly shown that there is a determined cut-off beyond which the chances of mortality increase. However, the same study also showed that there was a difference in the mean NLR in various studies conducted in different geographical locations [25].

A systematic review by Kuikel et al. (2022) also similarly examined the role of NLR as a predictor of adverse outcomes in CAP patients and noted that the cut-off value of more than 10 has shown predictive ability regarding mortality among CAP patients. In the present study, this cut-off value of NLR has been found to be 16 [26]. The present study further determines the capability of NLR as a mortality as well as severity marker among CAP patients admitted to the hospital. 

Decision-wise, it is preferable to use the NLR to recognize the likelihood of possible adverse outcomes at the bedside. In our cohort of patients with intermediate risks as specified by CURB-65, an NLR points to a higher need for ventilatory support and ICU admission. Although patients with major comorbidities that could affect disease severity were excluded as per exclusion criteria, a significant proportion of patients belonging to the geriatric population could have contributed to the high rate of ICU admission and Long ICU stay.

Similarly, it was observed that an NLR of at least the limiting number of 10 identified a group of hospitalized CAP patients who required increased ventilatory support and ICU need, warranting further attention, prompt upgrading, or specific scrutiny of antimicrobials and supportive care. On the contrary, when the NLR is low, and the remainder clinical parameters are good, ward-level care with close follow-ups could be sufficient for CAP patients. Since the NLR is a dynamic parameter, a physician may theoretically include the trajectory of rise/fall over 24-48 hours in both the disposition and the antimicrobial stewardship decision-making. The role of the NLR becomes more important as culture positivity among hospitalized CAP patients is often low, as observed in previous comprehensive analysis, where the diagnostic yield of routine blood culture in CAP ranges between 0% and 14% [27].

In our study of CAP patients, a high proportion of blood/sputum cultures were found to be sterile (85%). Our observation was found to be consistent with a prior study, which showed that even when optimal sampling and advanced diagnostic stewardship (induced sputum or repeated sampling) are applied, the etiological confirmation is achieved in only about a quarter to a third of hospitalized CAP patients [28]. This can have operational appeal, and blood NLR can be utilized in the diagnostic workup as well as in considering antibiotic upgradation among CAP patients admitted to the hospitals, particularly in low-resource countries. 

Multiple advantages support the use of the NLR as a prognostic indicator in CAP. It is easy to measure, has a low cost in determination, and a strong correlation with established severity scores [25,26]. All these advantages make the NLR a practical tool for early triage, risk assessment, and clinical decision-making, particularly in settings with limited access to advanced diagnostics. The identified threshold values can also be utilized to provide region-specific reference points for clinicians.

Limitations of the study

Being a single-center study, the findings may not be extrapolated to other populations. The exclusion of immunocompromised and pregnant patients may limit the applicability of results. Furthermore, a direct comparison of the NLR with other biomarkers was not performed, which could have strengthened the findings of the index study. Testing for atypical pathogens such as Legionella and Mycoplasma would certainly have strengthened the microbiological profile; however, these investigations are not routinely available in our institution and were not performed in all our patients during the study period. Absence of ROC-based justification for NLR cut-offs was another statistical limitation of our study. 

## Conclusions

The NLR has been used as an adjunct to the mainstream prognostic tools for assessing CAP. Furthermore, it closely correlates with CURB-65 and PSI scores and may be considered an accurate indicator of CAP severity. Integration of the NLR with clinical scores like CURB-65 and PSI may eventually lead to more accurate and accessible pneumonia risk stratification models for real-world use. Studies related to geographical location and population groups are important to determine regional and national cut-offs for prognostic scores of NLR among CAP patients. Further investigations in larger and more diverse populations are recommended to determine widely acceptable reference ranges and cut-offs of the NLR in CAP.
